# No Moral Wiggle Room in an Experimental Corruption Game

**DOI:** 10.3389/fpsyg.2021.701294

**Published:** 2021-08-18

**Authors:** Loukas Balafoutas, Fedor Sandakov, Tatyana Zhuravleva

**Affiliations:** ^1^Department of Public Finance, University of Innsbruck, Innsbruck, Austria; ^2^Higher School of Economics University, Moscow, Russia

**Keywords:** information avoidance, corruption, negative externality, experiment, Russia, C91, D73, D91

## Abstract

Recent experimental evidence reveals that information is often avoided by decision makers in order to create and exploit a so-called “moral wiggle room,” which reduces the psychological and moral costs associated with selfish behavior. Despite the relevance of this phenomenon for corrupt practices from both a legal and a moral point of view, it has hitherto never been examined in a corruption context. We test for information avoidance in a framed public procurement experiment, in which a public official receives bribes from two competing firms and often faces a tradeoff between maximizing bribes and citizen welfare. In a treatment where officials have the option to remain ignorant about the implications of their actions for citizens, we find practically no evidence of information avoidance. We discuss possible reasons for the absence of willful ignorance in our experiment.

## Introduction

As with many types of criminal activity, individuals who are prosecuted by the law due to corruption sometimes argue that they were not aware of corrupt activity taking place, or at least that they did not knowingly participate in such activity. The possibility that someone is not aware of the harm that he or she creates is relevant from a legal, but also from a moral point of view: In particular, virtue and deontological ethics base their value judgments not on the consequences of an action, but on the action itself or on the character of the person who takes it. However, having no positive knowledge of a corrupt act does not necessarily exonerate an individual. An important and pertinent question is, *could that individual have known* of the wrongdoing in question? In 1977, the US Congress enacted the Foreign Corrupt Practices Act (FCPA), which stipulates that knowledge of a corrupt activity goes beyond actual knowledge and extends to conscious disregard, deliberate ignorance, and willful blindness. This means that individuals who willingly ignore indications of wrongdoing in their area of responsibility—despite believing that a high probability of wrongdoing exists—can face criminal liability in cases of bribery and corruption^1^.

The above considerations motivate us to ask the following question in the present study: Do decision makers create moral wiggle room by choosing to remain blind to information in a corruption context, even though this information is potentially critical in distinguishing between corrupt (but privately profitable) and non-corrupt actions? A second, related question is whether corruption becomes more frequent when the possibility to engage in willful blindness is present—in other words, whether moral wiggle room is exploited. Moreover, motivated by the large costs imposed by bribery and corruption on third parties and by the open debate in the literature regarding the role of negative externalities for corrupt activities, we search for behavior consistent with moral wiggle room exploitation under different levels of negative externalities created by corruption.

We test for the presence of willful ignorance by decision makers in a lab experiment, which is meant to capture essential elements of a corruption setting. The decision situation in the experiment mimics a case of public procurement, where firms compete for a government contract. A *public official* purchases a service from a firm, and two competing *firms* may bribe the official in order to win the contract. There is also a *citizen* whose payoff is determined by the performance of the firm that wins the contract. We use framed and loaded instructions, in order to ensure that participants better understand the nature of the interaction and to enhance the ecological validity of our findings^2^. Experimental bribery games with participants in the role of decision makers (e.g., public officials), firms, and—sometimes also—affected third parties are very common in the literature on corruption (for a survey, see Abbink and Serra, 2012). Our experimental setting is similar to the corruption game used in Jaber-López et al. (2014), Schram et al. (2019), and García-Gallego et al. (2020). One notable difference is that the externality of corruption in our experiment is endogenous and determined through the performance difference between the two firms who compete for the government contract^3^.

Corruption is as widely prevalent around the world as it is costly (Svensson, 2005). In recent years, a growing body of literature has departed from neoclassical models of crime-and-punishment calculations that model corruption as the outcome of expected payoff maximizing calculations by economic actors (such as the seminal works by Becker, 1968 or Klitgaard, 1988). In addition, given the illegal nature of the phenomenon, reliable observational data on corruption are often hard to obtain, which in turn has led to a surge in research using data from the economic lab. This recent literature has offered experimental evidence on several (behavioral) aspects relating to corruption, such as social norms and culture (Cameron et al., 2009; Barr and Serra, 2010; Salmon and Serra, 2017; Schram et al., 2019), gender (Alatas et al., 2009), monitoring and punishment (Abbink et al., 2002; Armantier and Boly, 2011; Serra, 2012; Ryvkin et al., 2017), wages and appointment procedures of public officials (Azfar and Nelson, 2007), legal immunity for bribe givers (Abbink et al., 2014), transparency (Khadjavi et al., 2017; Parra et al., 2019), audience effects and observability (Salmon and Serra, 2017; García-Gallego et al., 2020).

Also relevant to our work are previous studies that have examined experimentally the role of negative externalities for the incidence of corruption and, in particular, the hypothesis that higher externalities should lead to lower levels of corruption, ceteris paribus. Interestingly, this is not always the case (Abbink et al., 2002; Büchner et al., 2008; Barr and Serra, 2009). Recently, Guerra and Zhuravleva (2021) examined the role of negative externalities and social norms in a corruption context. The focus of that study lies not on corrupt behavior *per se*, but on the willingness of unaffected bystanders to engage in third-party punishment of corrupt activities. Guerra and Zhuravleva (2021) find that bystanders are unresponsive to the variation in the negative externality, while Guerra and Zhuravleva (2020) report that female bystanders increase punishment when the externality goes up, while male bystanders decrease it. Overall, the effect of externalities on corruption remains an open research question that our work contributes to.

The possibility that decision makers exploit moral wiggle room in order to engage in more corrupt activities has hitherto not been examined in the economic literature, but it is related to a body of research reporting that participants in experiments involving distributional decisions often willfully avoid information regarding the consequences of their actions (e.g., Konow, 2000; Dana et al., 2007; Kajackaite, 2015; Grossman and Van der Weele, 2017; Regner, 2018). Broadly speaking, the existence of moral wiggle room allows decision makers to increase their monetary income by means of more selfish actions at the cost of other individuals, without incurring too high losses in terms of social image and self-image. In our experiment, we apply this notion to a corruption game. The extent to which individuals create and exploit moral wiggle room is a question of particular importance in the case of corruption, given the high societal costs associated with corrupt activities. Moreover, while it seems clear that the social norm in settings such as the dictator game involves at least some degree of pro-social behavior (Krupka and Weber, 2013) and selfish actions can produce high moral costs in the absence of moral wiggle room, the social norm in bribery games is not necessarily as well-established. Depending on the cultural background and broader context, bribe maximizing behavior may not represent a severe norm violation in some cases, reducing the need for bribe taking individuals to engage in willful ignorance in order to preserve their self-image. If this is true, willful ignorance may be less relevant in the context of corruption.

Information avoidance and the exploitation of moral wiggle room can be viewed as part of a larger literature on motivated reasoning, which refers to the idea that individuals avoid, distort, or misinterpret information in order to maintain a certain set of beliefs, from which they draw positive utility. The only study we are aware of that examines motivated reasoning in a corruption setting is Di Tella et al. (2015), who find that dictators are more likely to believe they are interacting with a dishonest recipient when they stand to gain more by behaving selfishly themselves. The corruption game used in Di Tella et al. (2015) is, however, very different from the one in our experiment, as it does not allow for information acquisition and essentially captures a case of embezzlement by an authoritarian ruler, while our game captures cases of collusive bribery featuring bribing firms, bribe-taking officials, and inactive but affected third parties. Thus, while both studies deal with corruption in a context of motivated reasoning, they refer to very different forms of corruption and institutional settings, measure different kinds of outcomes, and approach the topic through different perspectives.

## Experimental Design

### The Public Procurement Game

The experimental setting features three roles: *Public officials, firms*, and *citizens*. At the beginning of each session, all participants are randomly assigned one of these roles and interact in groups of four, consisting of one official, two firms, and one citizen. Participants keep their roles, but groups are re-shuffled in each round using a perfect stranger matching protocol. All groups play five rounds of the game described below, and one round is randomly selected and paid out at the end of the experiment^4^. At the beginning of the game, officials and firms receive an initial endowment of 10 ECU (where 1 ECU = 30 Ruble). Citizens receive no endowment.

**Figure 1:**
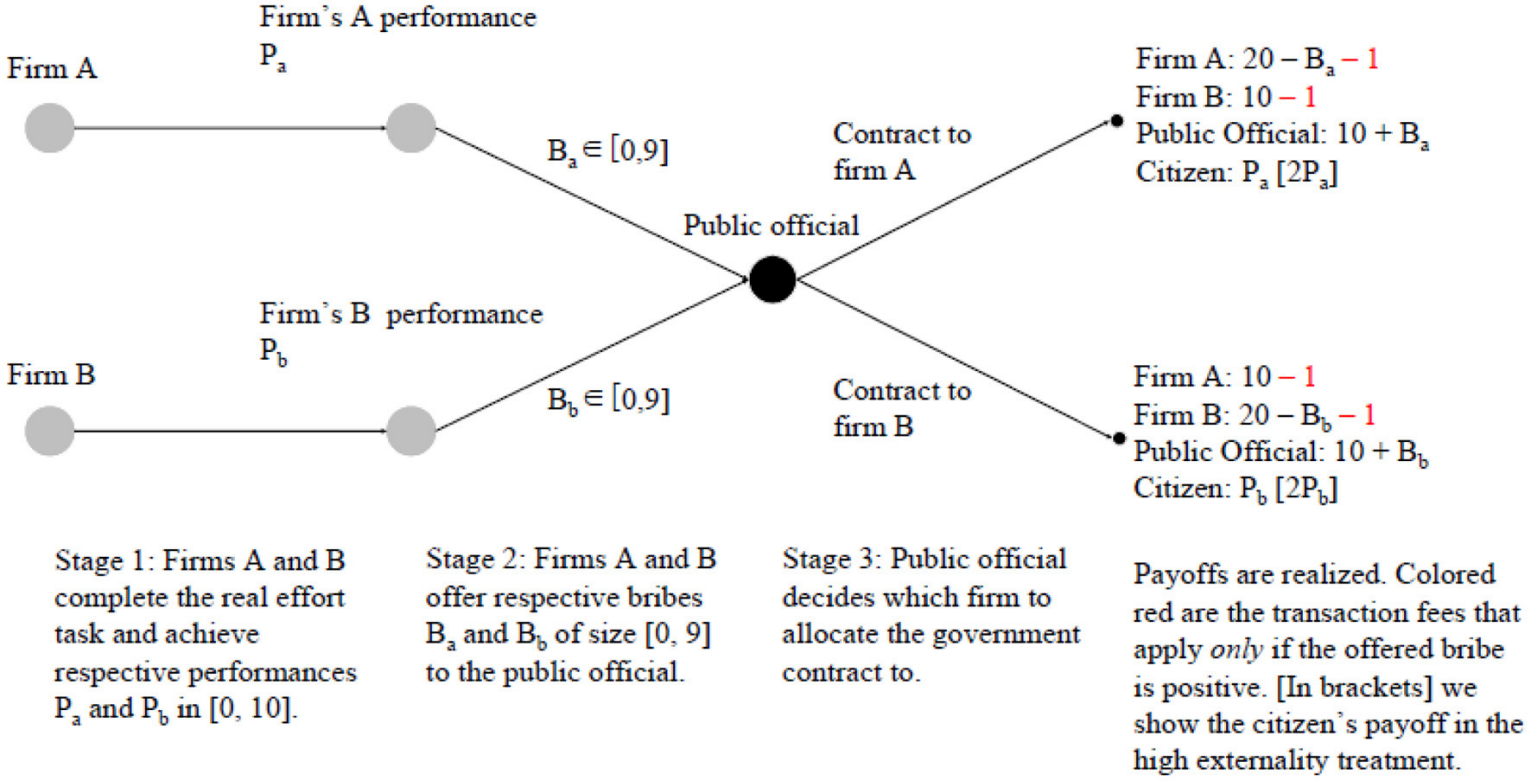
The game tree.

Figure 1 graphically represents the stages of the game. At Stage 1, each firm carries out the real-effort task used in Weber and Schram (2017), which consists of adding numbers and is described in section The Real-Effort Task. Each firm achieves a performance, which can vary between 0 and 10. This performance determines the payment of the citizen in the following way: The performance of the firm that wins the contract is multiplied by a factor of either 1 or 2 depending on the treatment (see section Treatments), and the resulting number is the citizen's income in ECU. Hence, notice that—in contrast to most other studies on corruption—the negative externality created by corruption is endogenous in this setting.

At Stage 2, each firm observes its own performance and has the opportunity to offer a non-negative amount (bribe) to the official out of their endowment. Every time a firm offers a bribe to the official, he or she pays an irrevocable transaction cost of 1 ECU, irrespective of the size of the bribe. This is meant to capture initiation costs of the briber when he approaches a public official (Abbink et al., 2002). Then, at Stage 3 of the experiment, the official receives the information about the firms' bribes and their performances and decides which firm wins the government contract. The winning firm receives an additional 10 ECU. The official keeps the bribe of the winning firm, while the bribe of the losing firm is transferred back to that firm.

An experimental session includes 24 participants. At the final stage of the experiment, participants complete a survey that includes basic socio-demographic information and a few questions from the World Values Survey (see Appendix D for a list of all survey questions). In addition to payoffs from the game, each participant receives 5 ECU if he or she completes the survey at the end.

### Treatments

The above description refers to the baseline treatment, which we call the *Full Information* treatment. In order to examine the presence of willful ignorance, we implement a further treatment with *Information Avoidance*. The only difference between the two treatments is at Stage 3, where the public official receives only the information about the two firms' bribes as a default option in the *Information Avoidance* treatment and has the option to also receive information about the firms' performance. This is implemented as follows: On his or her decision screen, the public official sees the two firms' bribes, and there is also a “Reveal performances” button that they can click on if they wish to. If they choose to click on that button, they see the two firms' performances on their screen.

In addition, we implement a variation in the size of the negative externality that is created by the official whenever he or she does not select the firm with the highest performance as the winner of the government contract. This is achieved by having one treatment with a *High Externality*, in which the performance of the winning firms is multiplied by a factor of 2 in order to determine the income of the citizen, and one treatment with a *Low Externality*, in which the performance of the winning firm is multiplied by a factor of 1. Hence, the experiment exploits a 2 × 2 treatment variation with four treatments in total, as shown in Table 1.

**Table 1:** Experimental treatments.

	**Full information**	**Information avoidance**
	***Treatment 1:***	***Treatment 2:***
High externality	Public official always knows firms' bribes and performances	Public official always knows firms' bribes and has the option to reveal performances
	Citizen's payoff = Performance of winning firm, multiplied by 2	Citizen's payoff = Performance of winning firm, multiplied by 2
	***Treatment 3:***	***Treatment 4:***
Low externality	Public official always knows firms' bribes and performances	Public official always knows firms' bribes and has the option to reveal performances
	Citizen's payoff = Performance of winning firm	Citizen's payoff = Performance of winning firm

### The Real-Effort Task

We use the real-effort task developed by Weber and Schram (2017) and used previously in corruption experiments (Schram et al., 2019; Di Zheng et al., 2020). On their computer monitor, firms see two 7 by 7 matrices filled with two-digit numbers. Their task is to find the largest number in each of the two matrices and add them up (see Figure A1 for an illustration). After entering their answer, a new set of randomly chosen matrices appears on the next screen, irrespective of whether the number entered was correct or not. This is an individual task and each firm has 3 min to solve as many of these matrix summations as they can. Firms' total performance in the task is a proxy for firm efficiency and determines citizen welfare in our setting. The maximum possible performance is 10, i.e., if a firm solves more than 10 matrices, its final performance is reduced to 10 (this was known to all participants). On the monitor, firms can see the remaining time and also the number of attempts and correct trials. At this stage, public officials and citizens wait.

### Hypotheses

While models of rational decision making predict that individuals would prefer to have more information when making decisions, the empirical literature has demonstrated that information is often avoided. A key explanation is that the lack of information serves as an excuse for selfish behavior. Specifically, Grossman and Van der Weele (2017) and Serra-Garcia and Szech (2019) show theoretically that willfully chosen ignorance is a compromise between material interest and a desire to maintain self-image. Empirical work confirms this claim, although the extent of this compromise varies with the setting and the parameters of interaction. For instance, in Van der Weele (2014) the share of participants who remain ignorant in a dictator game varies between 6 and 31%, depending on cost and benefit parameters. Grossman (2014) shows that the share of dictators who remain ignorant depends crucially on whether ignorance is an act of commission or omission. Recent literature explores willful ignorance in altruistic punishment (Kriss et al., 2016; Stüber, 2019) and shows that approximately a third of participants decide to remain ignorant about selfish dictators, in order to avoid the costs of punishing them. Felgendreher (2018) finds very little evidence for willful ignorance in a very different context (purchase of ethically certified products) compared to distributional games typically played in the economic lab.

Summing up the findings in previous literature, they have generally shown that willful ignorance is common, but it is also sensitive to the conditions and consequences that come with it. It is thus important to determine the extent to which people make a trade-off between material interests and self-image in a corruption context. Our first pre-registered hypothesis is that officials will exploit opportunities to avoid information and follow the selfish strategy more often.

H1:More officials will choose to maximize bribes in the treatments with information avoidance than in those without.

Our design additionally allows us to examine the role of externalities for corrupt behavior. We expect that, as long as (at least some) officials are concerned about the well-being of citizens, doubling the size of the externality will lead to more frequent choices that maximize citizen welfare. This is motivated by the extant experimental literature showing that individuals are driven by pro-social motives, including a taste for efficiency (see, e.g., Charness and Rabin, 2002; Engel, 2011). More specifically to a corruption context, higher externalities have sometimes been shown to reduce bribery (Barr and Serra, 2009).

H2:The share of officials who choose to maximize bribes is decreasing in the level of the externality.

On the other hand, the results on the effects of negative externalities on bribing behavior are mixed, and neither Abbink et al. (2002) nor Büchner et al. (2008) find any relationship between the level of externality and individuals' decisions in a public procurement context. We thus present and test H2 as it has been pre-registered, while keeping in mind that the literature on which it is based is rather inconclusive.

Our third hypothesis is based on H1 and H2 and essentially captures an interaction thereof. If officials deliberately avoid information as a way of justifying more selfish choices (H1), and if they tend to make fewer selfish choices in the presence of a high externality (H2), then it follows that the incentive to avoid information is weaker when the externality is higher, ceteris paribus. It should be noted, though, that in the literature there is little evidence that the loss of the other party affects the propensity of individuals to exploit moral wiggle room. For instance, in Van der Weele (2014) it is shown that the size of others' potential benefit has little effect on willful ignorance.

H3:Officials will choose to reveal more information on firms' performance when the level of the externality is higher.

### Procedures

We conducted the experiment in March 2021, with 20 sessions (five for each treatment), following a pre-registration that specified the hypotheses, procedures, sample size, and data analyses^5^. This led to a sample size of 120 participants per treatment, which includes 30 public officials and thus 150 observations for officials' decisions per treatment, given that they play five rounds of the game. We note, however, that we define each official as one independent observation in the statistical analysis, given that the five decisions by an official are not independent of each other.

The experiment was run at the HSE University in Russia and all participants were students of that institution. They were recruited through the manager of each educational program at HSE University (there are about 100 programs in four campuses in Moscow, St Petersburg, Perm, and Nizhny Novgorod), who was contacted and asked to send an e-mail to all students in his or her program. More than half of the managers agreed to do so. In this e-mail, we informed students about the study, their potential payoffs and asked them to fill out a google form with a convenient time slot. Nine hundred and sixty nine students filled out the form. Then we randomized these students across treatments, respecting their time preferences, and sent the invitation to a Zoom meeting to 45 students for each session (while only 24 were needed). Overbooking was necessary, since about 25 out of 45 students showed up for the meeting at a given time. We also had some “reserve” participants to ensure full sessions if fewer than 24 students appeared^6^. Each session lasted ~70 min in total, including reading the instructions and answering questions. The average earnings were 500 RUR (about 6.5 US dollars) per participant, which exceeds the average hourly wage in Russia.

The experiment was computerized using oTree (Chen et al., 2016) and we deployed the game online using Heroku services. The study was conducted via Zoom: Each participant received an invitation and, as soon as all participants were connected, the experimenter distributed individual links to oTree and read the instructions aloud (sample instructions are provided in Appendices A, B, C). In case of questions, participants could ask the experimenter directly or via the Zoom chat. Participants were asked to disable videos in Zoom in order to ensure confidentiality. To make sure that all participants understood the instructions, a computer-based quiz with four comprehension questions was conducted before starting the experiment, with direct feedback and explanations in case of an incorrect answer. About 75% of participants answered all four questions correctly on the first try^7^. The same experimenter conducted all 20 sessions, for consistency and to ensure that differences across sessions and treatments could not be attributed to experimenter-specific characteristics.

A total of 480 students participated, 34.5% of whom were male, and with a mean age of 21 years. Each subject participated in only one session. Ninety five percentage of participants were Russian by nationality and the remaining 5% came from post-Soviet republics (Belarus, Kazakhstan, Moldova, Latvia, Uzbekistan, and Ukraine). Only four out of 480 participants were married and only one had children. Most participants were undergraduate students (90%), 9% had a Bachelor degree. Thirty five percentage defined themselves as Christians, 55% as atheists, the other 10% were other denominations. Table A1 presents descriptive statistics, for the whole sample and separately by information avoidance and full information treatment^8^.

## Results

We report experimental results on *corruption choices* and *information choices* of public officials. To define and measure corruption, we record whether officials award the government contract to the firm with the highest performance or maximize bribes instead^9^. Information choices refer to the question of whether officials choose to reveal the information regarding the performances of the two firms when given the option.

Since we have five decisions per official (one for each round in a session), the main variable used in the data analysis, which we will be calling *share*, is the number of cases in which the official in a group took a bribe-maximizing decision during the course of the five rounds of interaction, as a share of the total number of *relevant* observations. Relevant refers to all cases in which an official faces a tension between bribe- and welfare maximization. This tension arises when the firm with the lower performance is the one that offers the higher bribe^10^. If, for instance, an official faces such a tension in four out of five rounds and chooses to maximize bribes in two of those cases and to maximize welfare in the other two cases, the value of *share* is 0.5 (= 2/4). In addition, choices in cases of ties in performance indicate only a weakly stronger concern (for bribe- or welfare maximization) by the official. We then employ two definitions: (i) *strict—*this includes cases when the official selects a firm with a higher bribe and lower performance, as a share of all cases when both bribes and performances are different (244 out of 600 officials' decisions in total), as well as situations when bribes are different and information on performances is avoided in the information avoidance treatments (25 out of 600 officials' decisions); (ii) *weak*—this definition includes all cases that fall under the definition of *strict*, adding situations when performances are equal (63 out of 600 officials' decisions). Situations with ties in bribes are excluded (65 out of 600 officials' decisions). This results in 112 independent observations on the variable *share* following the strict definition of bribe-maximizing behavior and 118 independent observations following the weak definition^11^.

**Figure 2:**
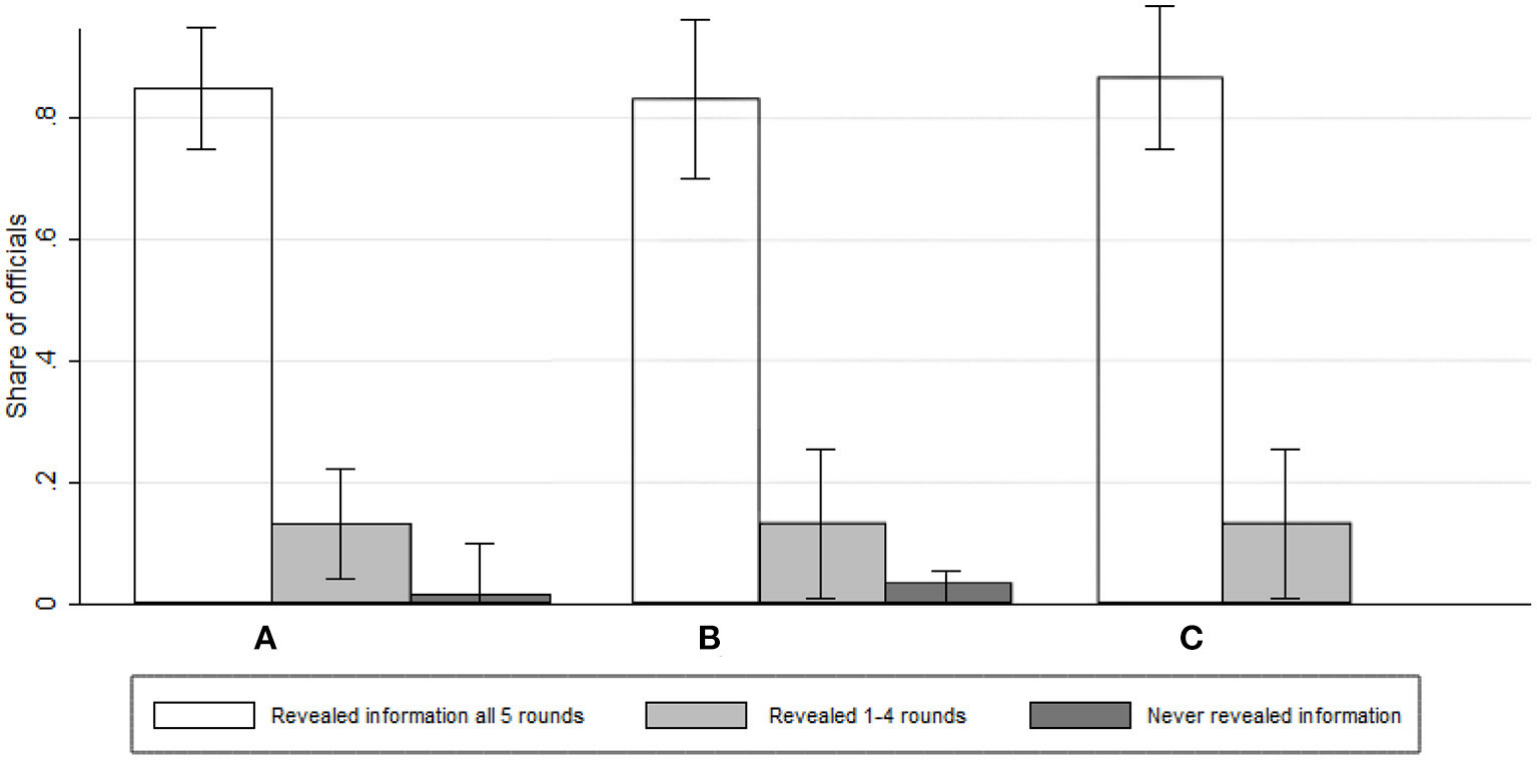
Information avoidance by officials. **(A)** Pooled. **(B)** High externality. **(C)** Low externality.

The departure point in our study is the question of whether public officials remain willfully ignorant in order to exploit a moral wiggle room. Hence, we begin the presentation of results by documenting information choices of public officials, i.e., whether they reveal the information on firms' performances when given the option to do so. We record the share of officials who reveal the information on firms' performance in the two information avoidance conditions (Treatments 2 and 4). Since we have five rounds and five observations per official per group, the outcome variable is the number of rounds (between 0 and 5) in which the official chose to reveal the performances of the two competing firms in a given group. The results are striking: In total, out of 300 decisions taken in total over the entire course of the interaction, officials decided to avoid the information about firms' performances only 25 times (8.3% of cases). This rate is much lower compared to previous studies that have endowed experimental participants with the opportunity to create and exploit a moral wiggle room^12^. Figure 2A below shows the share of officials who revealed the information in all five rounds, those who revealed it in at least one and at most four rounds, and those who never did. We observe that the overwhelming majority of officials never avoided the information.

Figures 2B,C allow us to test H3, by comparing the share of officials who choose to reveal information across treatments. In line with the graphical impression, a Mann-Whitney U test shows that the share of rounds (out of five) in which public officials chose to reveal information does not differ significantly between the low and the high externality conditions (0.93 vs. 0.91; *z* = −0.36; *p* = 0.72; *N* = 60).

***Result 1*. ***In the majority of cases, public officials always reveal information about firms' performances. This is true regardless of the level of externality, leading us to reject H3*.

Our test of H1 amounts to comparing the share of officials who maximize bribes across treatments, shown in Table 2. This comparison reveals that the difference between the *Full Information* and *Information Avoidance* treatments goes in the direction predicted by H1, but it is very small and insignificant, with bribe maximization rates of around 50% in both cases following the strict definition and roughly 60% following the weak definition (*strict*: *z* = 0.71, *p* = 0.48, *N* = 112; *weak*: *z* = 0.35, *p* = 0.73, *N* = 118; Mann-Whitney U tests). We also consider comparisons disaggregated by the level of externality: *share* does not differ by information treatment, under the low externality (*strict*: z = 0.72, *p* = 0.48, *N* = 59; *weak*: *z* = 0.17, *p* = 0.86, *N* = 60), or under the high externality (*strict*: *z* = 0.23, *p* = 0.82, *N* = 53; *weak*: *z* = 0.26, *p* = 0.80, *N* = 58; Mann-Whitney U tests).

**Table 2:** Mean *share*, by treatment.

	**Full information**		**Information avoidance**		**Overall**	
	**Strict**	**Weak**	**Strict**	**Weak**	**Strict**	**Weak**
High externality	0.55 (0.42)	0.65 (0.38)	0.59 (0.38)	0.69 (0.31)	0.57 (0.39)	0.67 (0.34)
Low externality	0.43 (0.38)	0.55 (0.34)	0.50 (0.41)	0.56 (0.34)	0.46 (0.40)	0.56 (0.34)
Overall	0.49 (0.40)	0.60 (0.36)	0.54 (0.40)	0.63 (0.33)	0.51 (0.40)	0.61 (0.34)
*N*	55	59	57	59	112	118

*Variable reports mean values of the variable share, as defined in the text. Standard deviations in parentheses. The number of observations (N) per condition is determined by the number of cases in which officials faced a tradeoff between bribe and citizen welfare maximization at least once*.

***Result 2*. ***Introducing the possibility of information avoidance has no effect on the inclination of public officials to maximize bribes over welfare. Hence, we reject H1*.

The rejection of H1 comes as no surprise, in light of the fact that public officials very rarely choose to remain willfully ignorant about the competing firms' performances. Result 1 essentially says that information avoidance is not a relevant phenomenon in the context of a bribery experiment such as the one considered here, as public officials generally do not create moral wiggle room for themselves. In line with this pattern, Result 2 states that bribe-maximizing behavior is independent of the presence of opportunities for information avoidance. We note, however, that out of the 25 cases in which public officials chose to remain willfully blind, they selected the higher bribe in 22 cases. Hence, while moral wiggle room is very scarcely created, those officials who do create it almost always exploit it.

To test H2, we compare the share of officials who maximize bribes in the two treatments with low externality (T3, T4) vs. two treatments with high externality (T1, T2). Bribe maximizing behavior is slightly more widespread under the higher negative externality: 46% of officials choose to maximize bribes when they face such a possibility (56% following the weak definition) in the low externality treatments, while in high externality treatments this share increases to 57% (67% following the weak definition). However, this difference is not statistically significant (*strict*: *z* = 1.38; *p* = 0.17; *N* = 112; *weak*: *z* = 1.85; *p* = 0.06; *N* = 118). We also consider disaggregated comparisons and test the choices of officials for each of the two information treatments separately. No significant differences are found^13^.

***Result 3*. ***When public officials face a tension between bribe- and welfare maximization, they maximize bribes in about half of such cases. The frequency of bribe-maximizing choices does not significantly vary by the negative externality imposed on the citizen, leading us to reject H2*.

In addition to the non-parametric analysis, we present in Table 3 a series of regressions in order to offer additional insights into the various factors that affect the behavior of public officials. In one set of regressions (columns 1–4, on officials' corruption choices), the dependent variable is *share* as defined above. The independent variables are the treatment dummies; interactions between treatments; the difference in the bribes offered by the two firms, computed as the sum of absolute differences in bribes in cases where officials face a tradeoff between bribe and welfare maximization divided by the number of such cases (as a measure of the monetary incentive to maximize bribes); as well as control variables from the post-experimental survey. In another set of regressions (columns 5–6, on officials' information choices), the dependent variable is the share of cases (out of 5) in which an official revealed the information on firms' performance. These regressions include the same set of independent variables as in the first four columns, except for the *Information Avoidance* treatment dummy (since information choices are only available in that treatment). All regressions are run using Ordinary Least Squares, with standard errors clustered at the session level.

**Table 3:** Regression analysis on public officials' choices.

	**Officials' corruption choice:** ***share***				**Officials' information choice**	
	**Strict definition**		**Weak definition**			
High externality	0.130 (0.122)	0.123 (0.113)	0.106 (0.109)	0.093 (0.096)	−0.020 (0.059)	−0.018 (0.066)
Information avoidance	0.086 (0.123)	0.140 (0.139)	0.016 (0.098)	0.054 (0.103)		
High externality × Information avoidance	−0.056 (0.155)	−0.036 (0.158)	0.019 (0.138)	0.050 (0.130)		
Difference in bribes	0.032^**^ (0.017)	0.035^**^ (0.020)	0.023 (0.018)	0.027 (0.022)	−0.012 (0.018)	−0.004 (0.022)
Control variables	No	Yes	No	Yes	No	Yes
Number of observations	112	112	118	118	60	60

*The number of observations in columns 1–4 is smaller than 120 due to the way the variable share is constructed: 8 and 2 officials (using the strict and weak definition, respectively) never faced a tradeoff between bribe and citizen welfare maximization. Standard errors, clustered at the session level, are in parentheses.*

** *denotes statistical significance at the 5% level*.

The regression analysis fully confirms Results 1–3. The level of externality affects neither the willingness of public officials to reveal information nor their propensity to maximize bribes. The option to remain ignorant about firms' performances (*Information Avoidance*) does not affect the corruption choices of public officials, and it does not interact with the externality level. As expected, a larger absolute difference in bribes—corresponding to a stronger motive for bribe maximization—is a significant predictor of an official's choice of the winning firm using the strict definition.

As a check of robustness, we estimate a set of regressions where the dependent variable is an individual official's round-by-round decision and use a random effects model to account for the interdependence of these decisions. Estimation results are given in Table A3. All previous results are fully confirmed: Neither the size of the externality nor the option to reveal information affects officials' willingness to maximize bribes. We observe that the absolute difference in the size of bribes (measured in a more accurate way case-by-case, compared to the sum of absolute differences in bribes used in Table 3) gains both in size and statistical significance. This variable becomes significant for the officials' information choice as well, with a negative coefficient. This confirms that the difference in the size of bribes is a strong motive for corruption.

Although firms are not the focus of our study, we briefly discuss some information about their behavior. Performances in the task display sufficient variation and vary from 0 to 10 matrices, with a mean of 4.59, standard deviation of 2.15, and median of 4 (see Figure A2). Similarly, bribes are offered in the entire possible range from 0 to 9 ECU, with a mean of 5.25, standard deviation of 2.61 and median of 6 (see Table A2 and Figure A3). Comparing across treatments, we confirm that randomization has been successful, since neither bribes nor performances differ significantly by treatment, either in the information or in the externality dimension^14^. We document a negative relationship between firm performance and bribes: The estimation of a linear regression model with bribe as the dependent variable yields a coefficient of −0.12 for performance (significant at the 5% level). This suggests that, on average, an increase of 8 points in performance leads to a one-unit reduction in the bribe.

**Figure 3:**
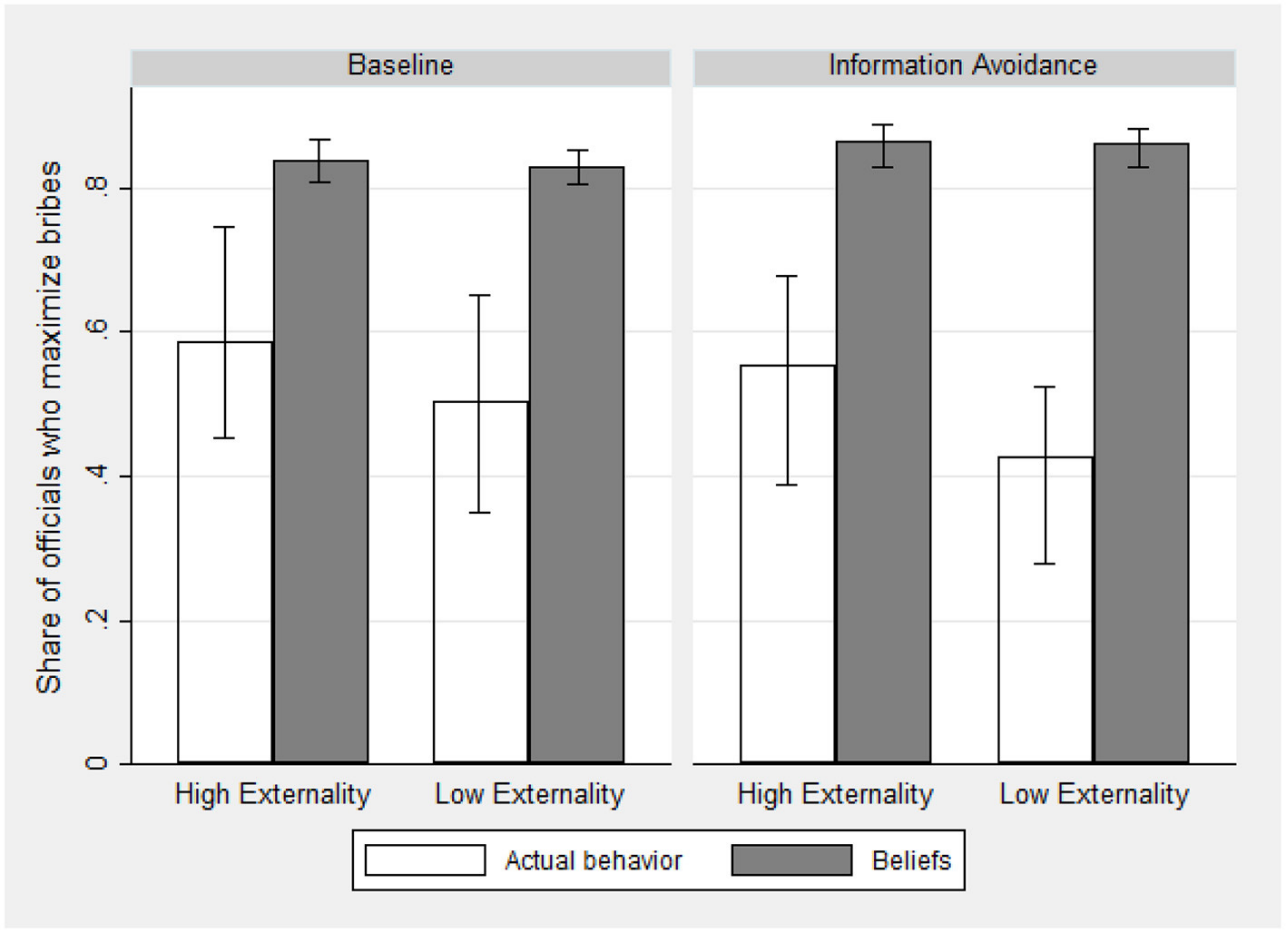
Bribe maximization: Beliefs vs. actual behavior, by treatment.

We also compare the beliefs of firms and citizens with the actual behavior of officials in order to reveal how successful they are in predicting the incidence of corruption. While officials were making their choices in Stage 3, firms and citizens were asked the following question: “*Out of the 7 officials*^15^ *in this session, how many do you think will choose a firm with a higher bribe instead of a firm with a higher performance, if they face such a tradeoff?*.” Interestingly, the difference between officials' actual bribe-maximizing behavior and firms' and citizens' expectations is pronounced. On average, across treatments, the share of officials who maximize bribes is 0.54, while firms and citizens expect it to be 0.85 on average. This pattern also holds if we make comparisons separately by treatments, see Figure 3. We find that this perceived frequency does not differ between treatments (0.83 in Full Information vs. 0.86 in Information Avoidance, *p* = 0.58, Mann-Whitney U test). The very high reported beliefs by firms and citizens point toward the absence of a descriptive norm against bribe taking, and it can help explain why officials do not create and exploit a moral wiggle room in our experiment. We return to this point in the discussion section.

## Discussion and Conclusion

Motivated by the legal and moral implications of willful blindness in settings of corruption, this study has examined the question of whether the widely documented phenomenon of information avoidance in economic experiments can be detected in a public procurement game. Our data deliver a negative answer: The majority (85%) of decision makers in the role of public officials obtain all relevant information in every round of the game when given the option to do so. Given this pattern, it is no surprise that we also document no differences in bribe taking behavior between the treatments with and without information avoidance opportunities. In addition, our study contributes to the open question on the role of negative third-party externalities on corruption: We find that bribe taking as a means of maximizing own payoff over citizen (and total) welfare is independent of the size of the negative externality.

The rejection of H1 and H3 is a consequence of the fact that public officials do not exploit opportunities for willful ignorance. The rejection of H2 is somewhat more surprising, although it fits well within the context of mixed findings in previous literature. At the same time, the rejection of all hypotheses may also be related to the small sample size of our study and to the way we define independent observations. While we have 590 (560) relevant observations on the behavior of officials following the weak (strict) definition of bribe maximizing behavior, our conservative testing procedure as defined in the pre-registration is based on only one-fifth of these figures. Minimum detectable effect sizes using this conservative procedure are as follows (referring here only to the weak definition in the interest of brevity): 0.19 for H1, 0.18 for H2, and 0.15 for H3, hence about one half of the observed standard deviations. This means that we cannot rule out the possibility that differences do exist across treatments in one or the other dimension, but they are smaller than the above figures and therefore not detectable in our study. This important caveat must be kept in mind and calls for replications of our results and additional evidence on the topic.

Why do participants in our experiment not avoid information? While we cannot give a definitive answer to this important question based on our dataset, we offer some thoughts on it. First, as noted in the introduction, ours is the first experimental study on information avoidance in a corruption setting. The fact that individuals have often been shown to avoid information in a self-serving manner does not mean that they will do so in every context. Corruption in the form of bribe payments (as implemented in our experiment) is a sensitive topic, widely discussed in politics and the media, and with far-reaching implications for society. When asked to place themselves in this situation (through the structure of the game and the loaded and framed instructions), experimental participants may find it important to have all available information at their disposal before they decide on a course of action. Their desire to make an informed decision for themselves and for the three other members of their micro-society may weigh in more than the motivation to maximize their own income without running the danger of compromising their (self-)image^16^.

Another possibility is that these findings are culture-specific. Our experiment was conducted in Russia, a country where corruption is very widespread. For instance, Mironov and Zhuravskaya (2016) reveals corruption in Russia by measuring the amount of cash channeled illegally out of firms around the time of regional elections and relating it to the probability that the firms obtained procurement contracts from the government. Zhuravleva (2015, 2021) shows that Russian households with workers in the public sector receive lower earnings than households with members employed in the private sector but enjoy the same level of consumption, and justifies this unexplained consumption-income gap by unreported income in the public sector. In 2020, Russia ranked only 129th out of 180 countries worldwide in the Corruption Perceptions Index published by Transparency International. In the 7th wave of the World Values Survey (WVS, see Haerpfer et al., 2020), respondents in Russia perceive corruption as very pervasive and the likelihood of being held accountable for corrupt practices as low. This question is also available for our sample. As it turns out, our participants are actually even more pessimistic than WVS respondents: In the question “*How would you place your views on corruption in your country*,” mean responses are 7.35 among WVS respondents and 8.95 in our sample (on a scale from 1 to 10, with higher values indicating higher perceived corruption). For comparison, the mean response in Germany (available during the same WVS wave and ranked at ninth place in the Corruption Perceptions Index) is 5.41.

In this context of high perceived and actual incidence of corruption, the moral costs of engaging in it are most likely substantially lower than in countries where bureaucrats are seen as more honest (see Balafoutas, 2011, for a theoretical model on public beliefs about corruption and how they shape the psychological costs for corrupt bureaucrats). Indeed, we have already shown in the previous section that the large majority of firms and citizens expect officials to maximize bribes when facing a tradeoff between bribes and citizen welfare. As a result, moral wiggle room is not as valuable, and much less often exploited. In terms of policy-related insights, this suggests that claims of ignorance often encountered in cases of corruption are quite unlikely to be true, and may be more often than not used as cheap talk or as an excuse by corrupt public officials. This would imply that such claims must be treated with particular skepticism by investigating authorities. Following these considerations, we believe that the replication of our study in countries with a lower incidence of corruption and strong anti-corruption norms would be a very interesting endeavor.

## Data Availability Statement

The raw data supporting the conclusions of this article will be made available by the authors, without undue reservation.

## Ethics Statement

The studies involving human participants were reviewed and approved by Board for Ethical Questions in Science of the University of Innsbruck. Written informed consent for participation was not required for this study in accordance with the national legislation and the institutional requirements.

## Author Contributions

All authors listed have made a substantial, direct and intellectual contribution to the work, and approved it for publication.

## Conflict of Interest

The authors declare that the research was conducted in the absence of any commercial or financial relationships that could be construed as a potential conflict of interest.

## Publisher's Note

All claims expressed in this article are solely those of the authors and do not necessarily represent those of their affiliated organizations, or those of the publisher, the editors and the reviewers. Any product that may be evaluated in this article, or claim that may be made by its manufacturer, is not guaranteed or endorsed by the publisher.

## Footnotes

^1^To mention one relevant example, in 2019, Former SNC-Lavalin CEO Pierre Duhaime pleaded guilty of helping a public official commit breach of trust, in a corruption scandal in connection with building a hospital in Quebec, Canada. He explicitly admitted to being willfully blind to the scandal and looking the other way, even though he was aware of corrupt actions inside his organization. Even though Mr. Duhaime was not shown to have actively engaged in the particular activity nor to have enjoyed any personal financial benefits from it, he was sentenced to 20 months in house arrest for remaining willfully blind to the scandal.

^2^The question of using neutral versus loaded instructions is debated in the experimental literature. The pioneering study of Abbink and Hennig-Schmidt (2006) does not find a significant difference between neutral and loaded framing in a bribery game. On the other hand, Ajzenman (2021) argues that people who observe that corruption is widespread are more willing to engage in corrupt behavior. Thus, using loaded instructions might have a different effect in different cultures. Our data are collected in one country, which means that the choice of loaded instructions is very unlikely to affect comparisons across treatments.

^3^The main reason for allowing firm performances to be determined endogenously by means of a real effort task has been to better capture real world settings, in which firms have a lot of discretion over the quality of their services. Another important feature of this setting is that it creates a variation in firm quality, without artificially imposing it.

^4^We let participants play five rounds of the game (instead of only one) because a tension between bribe and welfare maximization not always exists. Having five rounds practically ensures that all officials faced such a tradeoff in at least some periods. We refer to this issue again in the results section.

^5^Three sessions per day were conducted on March 13, 18, and 30; two sessions per day were conducted on March 16, 17, 19, 29, and 31; and one session was conducted on March 14. All the treatments were alternated between sessions.

^6^All reserve participants were HSE students as well, and most of them were students of one of the authors. In the end, the participation of a reserve participant was needed in only four cases.

^7^We have replicated all data analyses presented in Section 3 only for those participants who answered all four understanding questions correctly. All results remain unchanged.

^8^The sample is balanced for all control variables except for gender and education. Neither gender nor education was a focus variable in our study, hence we did not use block randomization along these dimensions. To account for the small observed imbalances, we control for gender and education (along with other control variables) in the regressions of Table 3. This does not affect any of the results and both variables are insignificant in the regressions.

^9^For ease of exposition we will be using the terms *corruption* and *bribe maximization* interchangeably, even though we acknowledge the fact that they do not perfectly overlap conceptually.

^10^When the official has information only on bribes, selecting the highest bribe without revealing performance is still classified as a bribe-maximizing decision.

^11^*Strict*: 26 observations in Treatment 1, 27 observations in Treatment 2, 29 observations in Treatment 3, 30 observations in Treatment 4. *Weak*: 29 observations in Treatments 1 and 2, 30 observations in Treatments 3 and 4.

^12^For instance, in the seminal study of Dana et al. (2007), 56% of participants chose to acquire costless information on relevant experimental parameters.

^13^*Full Information*, strict definition: z = 1.18; *p* = 0.24; *N* = 55; *Full Information*, weak definition: z = 1.15; *p* = 0.25; *N* = 59; *Information Avoidance*, strict definition: z = 0.73; *p* = 0.47; *N* = 57; *Information Avoidance*, weak definition: z = 1.37; *p* = 0.17; *N* = 59.

^14^Bribes: *z* = 0.59, *p* = 0.56 comparing *Full Information* vs. *Information Avoidance*, and *z* = −0.91, *p* = 0.36, *High Externality* vs. *Low Externality*. Performances: *z* = −1.46, *p* = 0.14 comparing *Full Information* vs. *Information Avoidance*, and *z* = −1.42, *p* = 0.16, *High Externality* vs. *Low Externality*. All tests reported here are Mann-Whitney U tests, treating average bribes or performances over all rounds within a group as one independent observation.

^15^We had 6 officials in each session but we asked about 7 in this question, due to an error. To compensate for this error, we computed the share for beliefs dividing the reported number by 7. This share can be compared against the share of corrupt choices by officials, dividing the number of bribe maximizing choices by the number of cases when officials faced a tradeoff.

^16^It must be noted that our game differs from most of the previous literature in one additional dimension (besides considering a different context and game): participants interact, and thus officials decide whether to remain ignorant, over five rounds. It might be harder to uphold a positive self-image when (knowingly) remaining ignorant for several times. If so, this feature of the design can help explain the very low incidence of information avoidance.
