# Supplementary material for: No Moral Wiggle Room in an Experimental Corruption Game
Source: Front Psychol. 2021 Aug 18;12:701294. doi: 10.3389/fpsyg.2021.701294 (PMC8416513; doi:10.3389/fpsyg.2021.701294)
Supplement: Supplementary file 1 [file Data_Sheet_1.docx]

**Appendix**

**A. Full instructions for Full Information treatment with High Externality (Treatment 1)^[[1]](#footnote-2)^**

Welcome to this experiment on decision-making and thank you for your participation! During the experiment, you and the other participants will be asked to take some decisions on your computer screen. The amount of money you earn in the experiment will depend on your decisions and on those of the other participants.

Please note that all your decisions will be entirely anonymous. You will never know anything regarding the identity of the participants you interact with, nor will we reveal anything about your identity to anyone else at any time.

All payoffs in the game are stated in ECU (Experimental Currency Units). At the end of the experiment, ECU will be converted to rubles at an exchange rate of 1 ECU = 30 Ruble.

The game consists of four stages, which we will now describe in detail. Stages 1-3 are repeated 5 times in different groups before stage 4 begins.

*The decision situation*

You will participate in a situation that mimics a case of public procurement, where two firms compete for a government contract. A public official who represents public authorities purchases a service from a firm, and competing firms may bribe the public official in order to win the contract. There is one more role in the group, a citizen, whose payoff is determined by the performance of the firm who wins the contract. At the start of the experiment, all participants are randomly assigned one of these roles and are randomly assigned to groups of four, consisting of one public official, two firms (Firm A and Firm B) and one citizen. An experimental session includes 24 participants, i.e. 6 groups.

At the beginning of the game, officials and firms receive an initial endowment of 10 ECU.

Citizens receive no endowment, and also make no decision whatsoever in the game.

At **Stage 1**, each firm independently carries out the real-effort task described below. Each firm achieves a performance for the task, which can vary between 0 (lowest performance) and 10 (highest performance). At this stage, public officials and citizens wait.

*The task performed by the firms*

You will see two matrices on the computer screen. Each matrix has 7 rows and 7 columns and is filled with randomly generated numbers. Your task is to find the largest number in each of the matrices and add them up. After entering the number, the computer will tell you whether it is correct or incorrect (the time will continue to run while you see the result). Then, irrespective of whether your answer is correct or incorrect, a new pair of matrices will appear. New matrices will appear as long as you are within the 3-minute limit. When the 3-minute limit ends, each firm will see the total number of correct solutions it has achieved. The maximum possible score is 10, so if your score exceeds 10, the excess will not be counted. For example, if someone solves 13 problems correctly, his or her score will still be 10. This number is its performance.

The performance of the firm that wins a government contract (see Step 3 below) **influences** **the payments of the citizen in that group**. This indicator is multiplied by 2 and becomes the citizen’s income. To clarify this point, consider an example in which a firm that won the contract correctly added 7 pairs of matrices in the task. The performance score 7 is multiplied by 2 and credited to the citizen in currency. His final gain is 7 ECU (from the performance of the winning firm) * 2 (coefficient) = 14 ECU.

At **Stage 2**, firms observe their performance (only their own performance, i.e. they do not know how they compared to the other firm in their group) and have the opportunity to offer an amount to the public official (out of their endowment of 10 ECU). Any amounts offered at this stage are referred to as bribes. Every time a firm offers a bribe to the official, he or she pays a transaction cost of 1 ECU, irrespective of the size of the bribe. This amount is simply debited from the firm’s account. The citizen is waiting at this stage.

At **Stage 3**, the public official receives the information about the two firms’ bribes and their performance and has to decide which firm wins the government contract. The winning firm receives an additional 10 ECU. The public official keeps the bribe from the winning firm, while the bribe of the losing firm is transferred back to that firm. The transaction costs of 1 ECU, however, are lost and not returned to the firms.

To help you understand the way payoffs are calculated, we offer here a randomly constructed example. Please go through this example carefully to make sure that you understand how the game works and how the payments are determined.

EXAMPLE: Firm A has a performance of 7 and offers a bribe of 4. Firm B has a performance of 5 and offers a bribe of 6. The official selects firm B as the recipient of the government contract. This means that the winning firm has a performance score of 5. The payments to group members after these decisions are:

Official’s payoff = 10 ECU (initial amount) + 6 ECU (bribe received from the winning firm) = 16 ECU.

Firm A’s payoff = 10 ECU (initial amount) - 1 ECU (non-refundable bribe transaction value) = 9 ECU.

Firm B’s payoff = 10 ECU (initial amount) - 6 ECU (bribe payment) - 1 ECU (non-refundable bribe transaction value) + 10 ECU (government contract) = 13 ECU.

Citizen’s payoff = 5 ECU (performance benefit of the winning firm) * 2 (coefficient) = 10 ECU.

The decision situation will repeat 5 times. Thereafter, you will go to stage 4.

**Stage 4: Questionnaire.** After completing steps 1-3 five times, you will be asked to answer a few questions about you and your preferences. If you complete this questionnaire, you will receive an additional payment of 5 ECU.

At the end of Stage 4, one round is randomly selected for payment and all participants are informed about their income from this experiment. This concludes the experiment. After receiving payments, you will need to send us a receipt of funds.

**B. Instructions for Information Avoidance treatments**

The only difference to the treatments with Full Information is that Stage 3 has the following instructions:

At **Stage 3**, the public official receives the information about the two firms’ bribes and **has the option** to also receive information about the firms’ performances. This is implemented as follows: on his or her decision screen, the public official sees the two firms’ bribes, and there is also a question ”Do you want to see the performances?”, public officials chose to click ”yes” or ”no” button. If they choose to click on the “yes” button, they see the two firms’ performances. Then, the public official has to decide which firm wins the government contract. The winning firm receives an additional 10 ECU. The public official keeps the bribe from the winning firm, while the bribe of the losing firm is transferred back to that firm. However, transaction costs of 1 ECU are lost and not returned to firms.

**C. Instructions for Low Externality treatments**

The only difference to the High Externality treatments is that Stage 1 has the following instructions:

The performance of the firm that wins the government contract (see Step 3 below) influences the payments of the citizen in that group. This performance becomes the citizen’s income. To clarify this point, consider an example in which a firm that won a government contract correctly added 7 pairs of matrices in a task. Citizen’s final gain becomes 7 ECU.

**D. Survey questions**

1. What is your year of birth?

2. What is your gender? (Male / Female )

3. What is your country of residence? (open question)

4. What is your nationality? (open question)

5. Are you married? (yes/no)

6. How many children do you have?

7. What is the highest educational attainment you have completed? (Elementary / High School / University Bachelor degree / University Master’s degree / PhD)

8. (This is Q171 from the WVS, on religiosity)

Apart from weddings, funerals and christenings, about how often do you attend religious services these days?

1 more than once week

2 once a week

3 once a month

4 Christmas/ Easter day

5 other specific holy days

6 once a year

7 less often

8 never, practically never

9. (This is Q57 from the WVS, on trust)

Generally speaking, would you say that most people can be trusted or that you need to be very careful in dealing with people? To indicate your views, use a 10-point scale where “1” stands for “need to be very careful” and “10” means “most people can be trusted”.

10. (This is Q112 from the WVS, on household income)

Consider an income scale, on which “1” indicates the lowest income group and “10” the highest income group in your country. We would like to know in what group your household is. Please, specify the appropriate number, counting all wages, salaries, pensions and other income.

11. (This is Q112 from the WVS, on the extent of corruption)

Now we’d like you to tell me your views on corruption: when people pay a bribe, give a gift or do a favor to other people in order to get the things they need done or the services they need. How would you place your views on corruption in your country on a 10-point scale where ”1” means “there is no corruption in my country” and “10” means “there is abundant corruption in my country”? If your views are somewhat mixed, choose the appropriate number in between.

12. (This is Q120 from the WVS, on accountability)

How high is the risk in this country to be held accountable for giving or receiving a bribe, gift or favor in return for public service? To indicate your opinion, use a 10-point scale where ”1” means “no risk at all” and “10” means “very high risk”.

13. (This is Q240 from the WVS, on political attitudes)

In political matters, people talk of “the left” and “the right”. How would you place your views on this scale, generally speaking? To indicate your views, use a 10-point scale where “1” stands for “Left” and ”10” means “Right”.

**E. Additional tables**

**Table A.1. Descriptive statistics for survey questions**

|  | Full sample | Information Avoidance | Full Information | *p* value |
| --- | --- | --- | --- | --- |
| Age | 20.72 (2.11) | 20.90 (2.68) | 20.55 (1.31) | 0.56 |
| Female | 0.65 (0.48) | 0.60 (0.49) | 0.70 (0.46) | 0.02 |
| Married | 0.01 (0.09) | 0.01 (0.09) | 0.01 (0.09) | 1.00 |
| Number of children | 0.01 (0.13) | 0.01 (0.19) | 0 (0) | 0.32 |
| Religiosity | 6.15 (1.27) | 6.14 (1.29) | 6.17 (1.26) | 0.89 |
| Education | 2.09 (0.32) | 2.13 (0.37) | 2.06 (0.25) | 0.02 |
| Trust | 6.33 (1.97) | 6.21 (1.90) | 6.44 (2.03) | 0.10 |
| Household income | 5.45 (1.30) | 5.50 (1.23) | 5.39 (1.37) | 0.41 |
| Extent of corruption | 8.95 (1.22) | 8.87 (1.31) | 9.03 (1.13) | 0.20 |
| Accountability | 4.80 (1.82) | 4.76 (1.73) | 4.85 (1.91) | 0.94 |
| Political attitudes | 4.87 (1.70) | 4.88 (1.63) | 4.86 (1.77) | 0.41 |
| Number of obs. | 480 | 240 | 240 |  |

*Notes*: Standard deviations in parentheses. Last column reports *p* values from χ^2^ tests for Female, Married and Education, and Mann-Whitney U tests for the remaining variables, comparing across the *Information Avoidance* and *Full Information* conditions.

**Table A.2. *Average bribe*, by treatment**

|  | Full Information | Information Avoidance | Overall |
| --- | --- | --- | --- |
| High externality | 5.15  (2.52) | 5.20  (2.62) | 5.17  (2.56) |
| Low externality | 5.29  (2.65) | 5.36  (2.66) | 5.33  (2.65) |
| Overall | 5.22  (2.58) | 5.28  (2.63) | 5.25  (2.61) |

*Notes*: The table reports mean values of the variable *average bribe*, as defined in text. Standard deviations in parentheses. The minimum possible bribe is 0 and the maximum is 9.

**Table A.3. Public officials’ choices: Random effects models**

|  | Officials’ corruption choices | | Officials’ information choices |
| --- | --- | --- | --- |
|  | Strict definition | Weak definition |  |
| *High Externality* | 0.101  (0.104) | 0.087  (0.087) | -0.022  (0.058) |
| *Information Avoidance* | 0.043  (0.100) | 0.007  (0.085) |  |
| *High Externality x Information Avoidance* | -0.055  (0.145) | 0.024  (0.121) |  |
| *Difference in bribes* | 0.065***  (0.013) | 0.048***  (0.012) | -0.019***  (0.005) |
| *Number of observations* | 269 | 332 | 300 |

Notes: The dependent variable in the first two specifications equals 1 if an official chose to maximize bribes in a given round (following the strict or the weak definition), and 0 otherwise. Officials’ choices in these specifications are estimated for the situations that included a trade-off between bribe and welfare maximization. The dependent variable in the third specification equals 1 if the official chose to reveal information in a given period, and 0 otherwise. Standard errors are in parentheses. *** denotes statistical significance at the 1% level.

**F. Additional tables**

**Figure A1. Screenshot of the real effort task.**


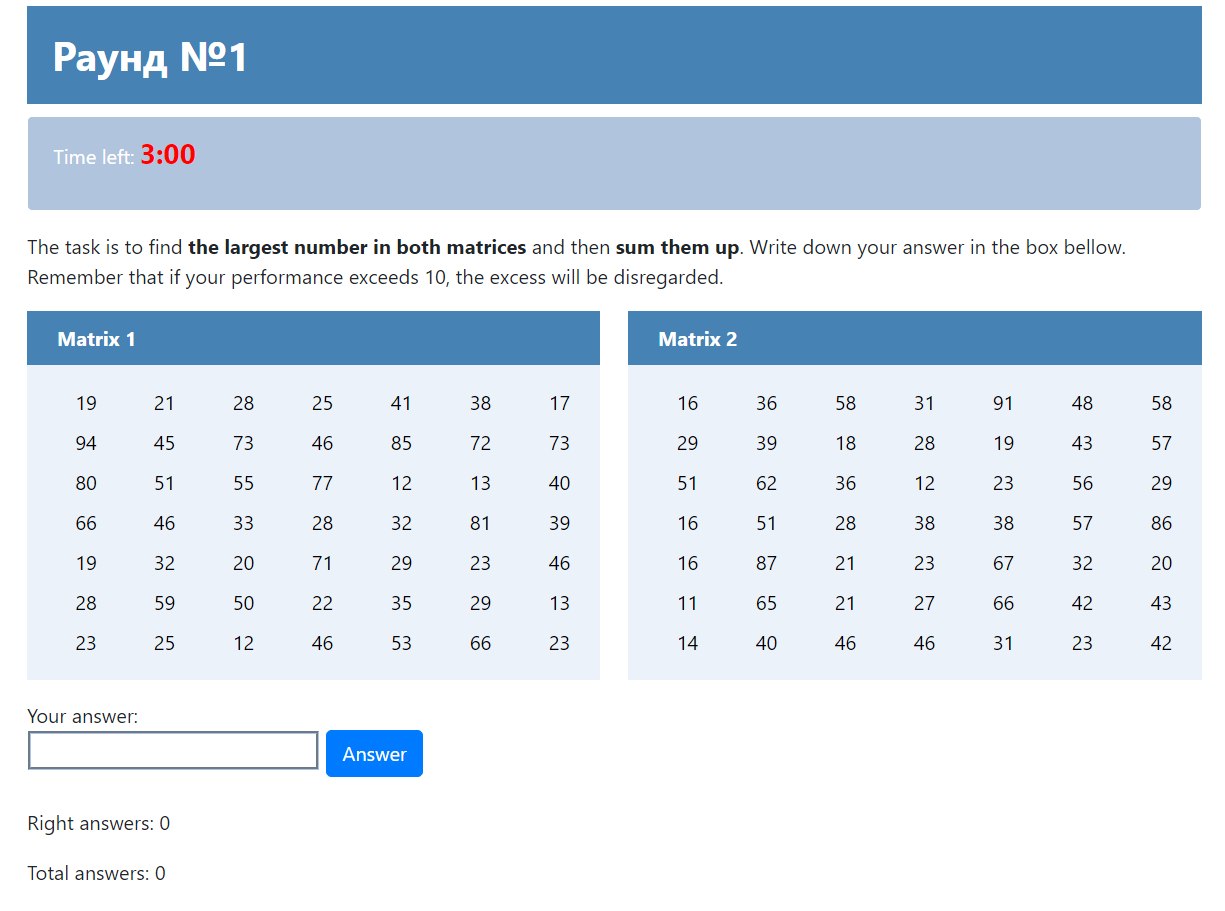


**Figure A2. Firms’ performances in the real effort task.**


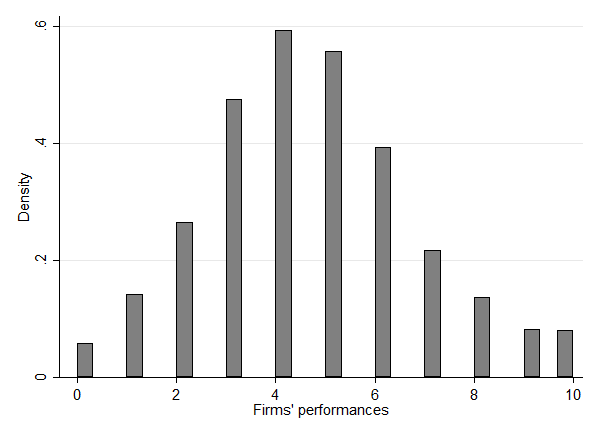


**Figure A3. Firms’ average bribes.**


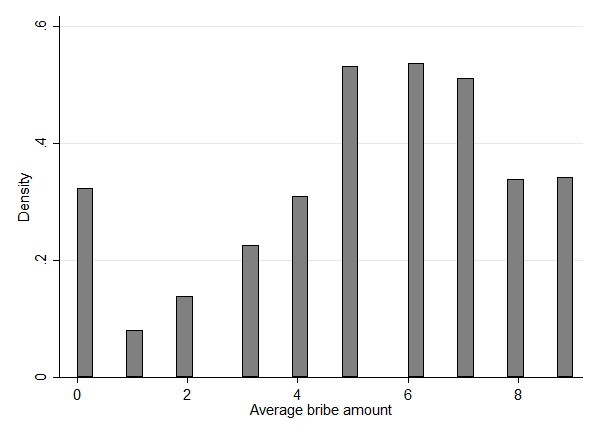


1. Original instructions in Russian are available from the authors upon request. [↑](#footnote-ref-2)
